# Differential changes in serum uric acid concentrations in sibutramine promoted weight loss in diabetes: results from four weeks of the lead-in period of the SCOUT trial

**DOI:** 10.1186/1743-7075-6-42

**Published:** 2009-10-14

**Authors:** Charlotte Andersson, Peter Weeke, Bente Brendorp, Lars Køber, Emil L Fosbøl, Arya M Sharma, Nick Finer, Ian D Caterson, Richard A Rode, Philip T James, Christian Torp-Pedersen

**Affiliations:** 1 Department of Cardiology, Gentofte University Hospital, Hellerup, Denmark; 2 Department of Cardiology, Glostrup University Hospital, Denmark; 3 The Heart Centre, University Hospital of Copenhagen, Rigshospitalet, Denmark; 4 University of Alberta, Royal Alexandra Hospital, Edmonton, Alberta, Canada; 5 Addenbrooke's Hospital, Institute for Metabolic Science, Cambridge, UK; 6 Institute of Obesity Nutrition & Exercise, University of Sydney, NSW Australia; 7 Abbott Laboratories, Abbott Park, Illinois, USA; 8 London School of Hygiene and Tropical Medicine, London, UK

## Abstract

**Background and aims:**

Elevated levels of serum uric acid are associated with an increased risk of cardiovascular morbidity and mortality. The response of uric acid to weight loss therapy (lifestyle plus sibutramine) in an overweight and obese cardiovascular high risk population was studied.

**Methods and results:**

Data from a four week single-blind lead-in period of the Sibutramine Cardiovascular OUTcomes (SCOUT) study were analyzed. 2584 patients (24%) had diabetes mellitus (DM) only, 1748 (16%) had cardiovascular disease (CVD) only and 6397 (60%) had both DM + CVD. Uric acid concentrations (mean ± standard deviation) at screening were significantly higher among patients with CVD compared to patients without CVD (p < 0.0001): 369 ± 86 μmol/L, 374 ± 98 μmol/L and 342 ± 87 μmol/L in CVD only, CVD+DM and DM only groups, respectively. During treatment uric acid decreased significantly more in patients without DM (p < 0.0001): -15.0 μmol/L (95% confidence interval -17.7;-12.4), -4.6 μmol/L (-6.2;-3.0), and -6.6 μmol/L (-8.7;-4.5) in CVD only, CVD+DM, and DM only groups, respectively. In patients who failed to lose weight, sibutramine induced lower uric acid levels, but greater weight loss and diabetes were associated with smaller falls in blood uric acid levels; decreasing fasting and urinary glucose concentrations in diabetes were associated with increases in uric acid levels.

**Conclusion:**

A four week daily intake of sibutramine and life style changes was associated with significant reductions in mean uric acid levels. Changes in renal glucose load in diabetes seem to counteract a potential uricosuric effect of sibutramine.

**Trial Registration:**

The trial is registered at ClinicalTrial.gov number: NCT00234832.

## Introduction

Large epidemiological studies of populations at increased risk of cardiovascular events (including those with diabetes, hypertension and a history of cardiovascular disease) show hyperuricaemia to powerfully predict cardiovascular adverse events and mortality[1-3], even within the high normal range of uric acid concentrations[4]. Hyperuricaemia may, however, simply reflect established cardiovascular risk factors, such as hypertension and renal impairment and not act causally[5].

Elevated serum uric acid levels, obesity, insulin resistance and type 2 diabetes mellitus frequently coexist[6,7] and weight loss with sibutramine reduces uric acid levels[8]. Whether this also occurs in patients at increased risk for cardiovascular adverse events and in diabetes mellitus is unknown. Therefore, we examined the uric acid response to short (four week) weight loss therapy with sibutramine in the overweight or obese, cardiovascular high risk population enrolled in the Sibutramine Cardiovascular OUTcomes (SCOUT) study.

## Methods

SCOUT is an ongoing randomized, double-blind, placebo-controlled clinical study of cardiovascular outcome in overweight or obese patients at increased risk for cardiovascular adverse events. Patients aged 55 years or older, with a body mass index (BMI) ≥ 27 kg/m^2 ^and ≤ 45 kg/m^2^, or a BMI ≥ 25 kg/m^2 ^and <27 kg/m^2 ^plus a waist circumference ≥ 102 cm if male and ≥ 88 cm if female, were included. A further requirement was either the presence of cardiovascular disease (CVD) (defined as a history of coronary artery disease, peripheral arterial occlusive disease or stroke), or diagnosed type 2 diabetes mellitus (DM) with at least one additional well defined risk factor (i.e. hypertension, dyslipidaemia, current smoker or diabetic nephropathy with evidence of microalbuminuria)[9]. Fifteen months after initial enrolment, inclusion criteria were modified due to a lower than expected overall primary outcome event rate. Thereafter, recruited patients were required to have both CVD and DM. All inclusion and exclusion criteria are described elsewhere[9]. Importantly, patients with more severe heart failure (i.e., greater than New York Heart Association (NYHA) class II; diagnosis was based on patient history and also requiring medical treatment) were excluded.

The present analyses deal with the first four weeks of the 6-week, single-blind, lead-in period of the SCOUT trial. The single-blind period preceded randomization into the main study, with all subjects receiving 10 mg sibutramine daily together with advice on diet and physical exercise. At the screening visit and every other week, a physical examination, including measurements of weight, height, blood pressure and pulse, was performed. Data on haematology and blood biochemistry were collected at the screening visit and after four weeks of treatment. All blood samples were taken fasting and analysed in a certified central laboratory. Some of the patients had a second haematology or blood biochemistry sample taken either in relation to the screening visit or in relation to the four week visit. For the present analysis, these secondary blood samples were used only if values from the first measurement were missing. Uric acid concentration had a normal reference defined as 150-450 μmol/L for women and 240-510 μmol/L for men. Creatinine clearance was calculated from measured creatinine concentrations, using the Modification of Diet in Renal Disease (MDRD) study equation[10].

All patients provided urinary samples that were semi-quantitatively (i.e., by dip stick) analyzed for ketone bodies, blood, proteins and glucose. For glucose, the dip stick scale spanned from "negative", "trace", "1+", "2+" to "3+". A one unit change of urinary glucose was defined as a change of one step on the scale (e.g., from trace to 1+, or from trace to negative).

The population was classified according to the presence of diabetes mellitus, and/or cardiovascular disease, using the inclusion diagnoses. Thirteen of 10,742 patients (0.12%) could not be classified in any of these groups because of missing data, and were therefore not included in the present analysis. In analyses stratified by diabetes, the diabetes group included the patients originally grouped in DM only group or in CVD+DM group. In analyses stratified by weight change, patients who maintained or gained weight were defined as one group and the other groups were composed according to each percentage of weight loss (i.e. lost >0-1%; lost >1-2%; lost >2-3%; lost >3-4%; and lost > 4% of their initial weight, respectively; further information and distribution of patients in the different weight change groups are available elsewhere[11]).

### Ethics

The study was performed in conformity with the Declaration of Helsinki and was approved by all relevant ethical committees. All patients gave their written, informed consent before participating. The trial is registered at ClinicalTrial.gov number: NCT00234832.

### Statistics

Continuous variables were compared by t-test and analysis of variance (ANOVA). Discrete variables were compared with chi-square test. All tests were repeated with non-parametric methods (rank sum test for continuous variables and Mantel-Haenszel chi square for discrete variables). Unless specified, results from the parametric models have been reported. General linear models were used to examine the impact of different factors on the change in uric acid concentration. Irrespective of significance levels, all variables with a possible influence on uric acid levels were included in multivariable analysis, as were the medications previously reported to influence uric acid levels[12]. However, measurements of the urinary glucose were not included, since the semi-quantitative scale tended to have a non-linear effect on the change in uric acid concentration. Unless otherwise mentioned, test for relevant interactions were found to be non-significant (p > 0.05).

Data available by September 2006 were used in all analyses. All calculations were made using SAS^®^, version 9.1 (SAS institute, Cary, North Carolina). The level of statistical significance was set at a p-value < 0.05.

## Results

The study included 10729 patients, of whom 2584 (24%) were classified as having only DM, 1748 patients (16%) as having only CVD, and 6397 patients (60%) as having both DM and CVD. The main characteristics of the different groups are shown in Table 1.

**Table 1:** Screening characteristics

	**DM only**	**CVD only**	**CVD+DM**
	**n = 2584**	**n = 1748**	**n = 6397**
**Age (years)**	62 (± 6)	64 (± 6)	64 (± 6)
**Male gender (%)**	38%	66%	64%
**BMI (kg/m^2^)**	35.9 (± 4.8)	33.4 (± 4.1)	34.0 (± 4.4)
**Weight (kg)**	97.4 (± 16.1)	95.0 (± 14.8)	95.8 (± 15.4)
**Waist circumference, men (cm)**	117 (± 12)	112 (± 10)	114 (± 10)
**Waist circumference, women (cm)**	111 (± 12)	104 (± 11)	109 (± 11)
**Systolic blood pressure (mmHg)**	141 (± 12)	136 (± 13)	138 (± 13)
**Diastolic blood pressure (mmHg)**	78 (± 8)	78 (± 8)	77 (± 9)
**Pulse (bpm)**	75 (± 10)	68 (± 10)	71 (± 10)
**Creatinine clearance (mL/min/1.73 m^2^)**	76 [48;105]	72 [47;99]	71 [41;103]
**Uric acid concentration (μmol/L)**	342 (± 87)	369 (± 86)	374 (± 98)
**Glucose concentration (mmol/L)**	9.0 (± 3.1)	6.0 (± 1.0)	8.9 (± 3.2)
**HDL cholesterol (mmol/L)**	1.3 (± 0.3)	1.2 (± 0.3)	1.2 (± 0.3)
**LDL cholesterol (mmol/L)**	3.1 (± 0.9)	3.0 (± 0.9)	3.0 (± 1.0)
**Triglycerides (mmol/L)**	2.2 (± 1.5)	2.0 (± 1.1)	2.4 (± 1.4)
**History of dyslipidaemia (%)**	71%	76%	86%
**History of stroke (%)**	0%	9%	12%
**Peripherial artery disease (%)**	0%	8%	16%
**Use of alcohol (%)**	51%	63%	54%
**Current smokers (%)**	11%	10%	9%
**Coronary artery disease (%)**	4%	87%	83%
**CHF (%)**	2%	9%	11%
**Use of diuretics (%)**	45%	37%	51%
**Use of Insulin (%)**	26%	0.5%	32%
**Use of statins (%)**	42%	73%	73%
**Use of ACE-inhibitors (%)**	73%	64%	82%
**Use of Ca^2+^-blockers (%)**	32%	33%	40%
**Use of fibrates (%)**	10%	6%	10%
*Urinary dip stick for glucose:*			
**Negative (%)**	81%	99%	79%
**Trace (%)**	6%	0.4%	6%
**1+ (%)**	4%	0.2%	5%
**2+ (%)**	3%	0.1%	3%
**3+ (%)**	6%	0.4%	6%

Continuous variables are presented as means (± standard deviation) and discrete variables as percentages (%). Creatinine clearance is presented as median [5^th ^95^th ^percentiles] due to widely distributed values. Blood samples were obtained during screening visit, as were medical histories. Weight, BMI, waist circumference, pulse and blood pressure are values from the first day of sibutramine administration.

At screening, uric acid concentrations were significantly higher among patients with a history of CVD, compared to patients with only DM (CVD group, compared to DM group: p < 0.0001 and CVD+DM, compared to DM group: p < 0.0001). Patients using diuretics had significantly higher uric acid concentrations, compared to patients not using diuretics (mean ± standard deviation [SD]: 388 ± 101 μmol/L, and 345 ± 84 μmol/L, respectively, p < 0.0001). Analyses stratified by use of diuretics also found CVD patients had higher uric acid concentrations, compared to non-CVD patients irrespective of diuretic use (data not shown).

**Figure 1:**
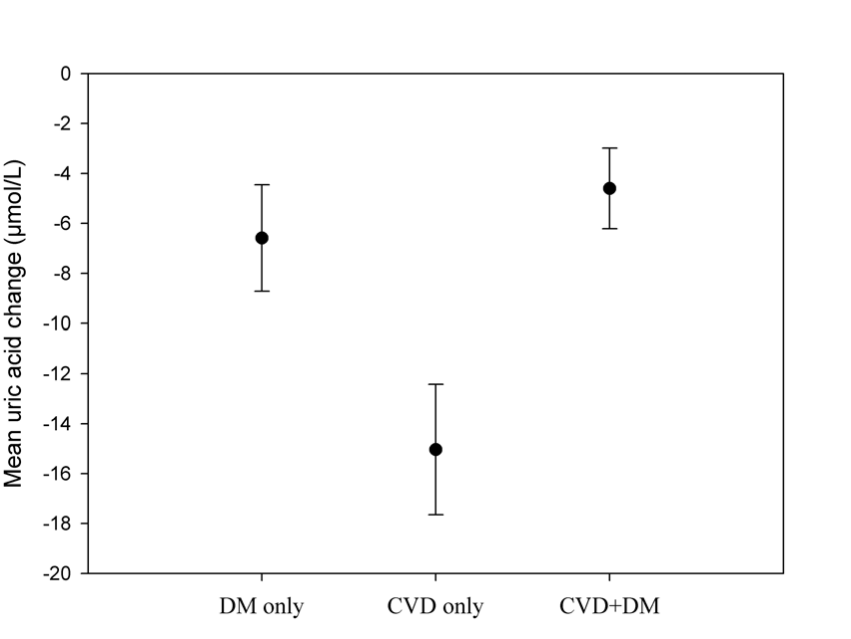
**Four week change in uric acid concentration, stratified for the presence of only diabetes mellitus (DM), only cardiovascular disease (CVD) or both (p-value for changes <0.0001 in respectively group)**. Error bars illustrate 95% confidence interval.

Over the first four weeks of sibutramine-based therapy, mean uric acid concentration decreased significantly in all three groups (Figure 1). Patients with diabetes had significantly smaller reductions in uric acid (DM only group, compared to CVD only group: p < 0.0001 and CVD+DM, compared to CVD only group: p < 0.0001). Mean weight loss was 2.2% (± SD 1.8), 2.1% (± 1.8) and 2.4% (± 1.9) in the DM only, CVD+DM, and CVD only group, respectively. Multiple factors were significantly associated with a four week change in uric acid concentration, as shown in the left column of Table 2. As indicated with (*) or (†) in Table 2, many of the factors were found to affect the change in uric acid levels differently in patients with and without diabetes.

**Table 2:** Variables with a significant effect on the four week change in uric acid. Results from multivariable regression analysis

	***All patients***		***Diabetes***		***No diabetes***	
	*Estimated influence on uric acid concentration (μmol/L):*	**p-value:**	*Estimated influence on uric acid concentration (μmol/L):*	**p-value:**	*Estimated influence on uric acid concentration (μmol/L):*	**p-value:**
Change in glucose concentration (for 1 mmol/L decrease) *	1.6 (± 0.3)	<0.0001	1.7 (± 0.3)	<0.0001	-3.8 (± 1.5)	0.01
Weight loss, for 1% decrease *	2.5 (± 0.3)	<0.0001	2.9 (± 0.4)	<0.0001	0.6 (± 0.7)	0.4
LDL change (for 1 mmol/L decrease) †	-7.4 (± 0.9)	<0.0001	-6.4 (± 1.1)	<0.0001	-11.7 (± 2.0)	<0.0001
Triglyceride change (for 1 mmol/L decrease)	-6.3 (± 1.1)	<0.0001	-6.0 (± 1.2)	<0.0001	-7.4 (± 2.6)	0.004
Change in systolic blood pressure (for 1 mmHg decrease)	0.3 (± 0.07)	<0.0001	0.3 (± 0.08)	0.0002	0.1 (± 0.1)	0.5
Use of fibrates	-14.7 (± 2.1)	<0.0001	-15.3 (± 2.2)	<0.0001	-8.9 (± 5.7)	0.1
Triglyceride concentration at baseline (for 1 mmol/L increment)	5.5 (± 0.9)	<0.0001	5.8 (± 1.0)	<0.0001	3.6 (± 2.2)	0.1
Use of diuretics	11.9 (± 1.2)	<0.0001	12.1 (± 1.4)	<0.0001	10.0 (± 2.9)	0.0007
Uric acid concentration at baseline (for 1 μmol/L increment)	-0.2 (± 0.01)	<0.0001	-0.3 (± 0.007)	<0.0001	-0.2 (± 0.02)	<0.0001
Creatinine clearance at baseline (for 1 mL/min/1.73 m^2 ^increment)	-0.08 (± 0.02)	<0.0001	-0.07 (± 0.02)	<0.0001	-0.2 (± 0.06)	<0.0001
Change in creatinine clearance (for 1 mL/min/1.73 m^2 ^increase)	0.1 (± 0.01)	<0.0001	0.1 (± 0.02)	<0.0001	0.2 (± 0.05)	<0.0001
Male gender	10.2 (± 1.4)	<0.0001	10.2 (± 1.6)	<0.0001	11.1 (± 3.5)	0.001
Waist circumference at baseline (for 1 cm increment)	0.2 (± 0.07)	0.001	0.2 (± 0.08)	0.007	0.2 (± 0.1)	0.1
BMI at screening (for 1 kg/m^2 ^increment)	0.5 (± 0.2)	0.01	0.5 (± 0.2)	0.02	0.5 (± 0.4)	0.3
HDL change (for 1 mmol/L decrease) *	5.7 (± 4.0)	0.2	10.0 (± 4.5)	0.03	-10.8 (± 8.3)	0.2
LDL at screening (for 1 mmol/L decrement)	0.3 (± 0.7)	0.6	0.8 (± 0.8)	0.3	-3.6 (± 1.6)	0.02
Type 2 diabetes mellitus	5.9 (± 1.7)	0.0004	-		-	-

The stratum "diabetes" includes the patients in the DM only group and the CVD+DM group. "No diabetes" includes the patients in CVD only group. Only variables at a significance level of ≤ 0.05 in overall group or in any of the two groups are presented in table. Analysis was also adjusted for screening features (age, smoking status, a diagnosis of heart failure, systolic and diastolic blood pressure, pulse, glucose concentration at baseline, and HDL cholesterol at baseline), medication use (insulin, aspirin, ACE-inhibitors, calcium channel blockers, statins) and dynamic variables (changes in diastolic blood pressure and pulse). SE = standard error. (*) and (†) indicate a p-value ≤ 0.01 and p-value ≤ 0.05 respectively for differences in the influence on uric acid between patients with and without diabetes.

### Diabetes status and the influence of weight loss and changes in fasting glucose on uric acid concentration

**Figure 2:**
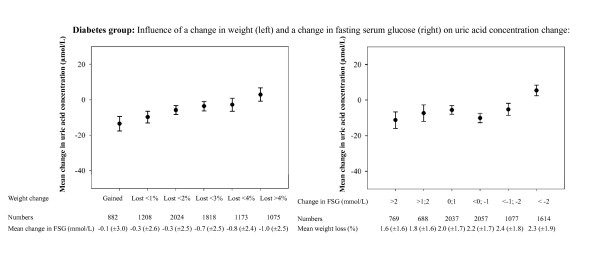
**Mean change in uric acid concentration, according to four week weight change (left) and four week mean change in fasting serum glucose (FSG, right) in patients with diabetes**. "Numbers" refers to the numbers of patients with available values on the uric acid change in the respective group. The mean uric acid concentration change was found to differ in both the groups of weight change and the groups of FSG change (p < 0.0001 in both analyses). Error bars illustrate 95% confidence interval.

**Figure 3:**
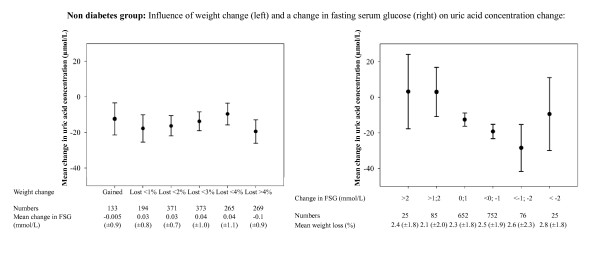
**Mean change in uric acid concentration, according to four week weight change (left) and four week mean change in fasting serum glucose (FSG, right) in patients without diabetes**. "Numbers" refers to the numbers of patients with available values on the uric acid change in the respective group. No difference was found in mean uric acid concentration change between the weight change groups (p = 0.3). The mean uric acid concentration change was found to differ over the groups of FSG change (p = 0.0004). Error bars illustrate the 95% confidence interval.

Figure 2 and 3 illustrate the influence of changes in weight and fasting serum glucose on uric acid levels in patients with and without diabetes, respectively.

In patients with diabetes, increasing weight loss with sibutramine and lifestyle changes was associated with smaller reductions in uric acid. Adjusted for multiple variables, each 1% reduction in weight was associated with a 2.9 (± standard error [SE] 0.4, p < 0.0001) μmol/L smaller fall in uric acid concentration. In patients without diabetes, weight loss had no detectable impact on the uric acid levels: a 1% reduction in weight was associated with a 0.6 (± 0.7, p = 0.4) μmol/L increase in uric acid concentration. The difference in impact of weight loss between patients with and without diabetes was statistically significant (p for difference in multivariable analysis = 0.0003).

Decreasing levels of fasting serum glucose were associated with different changes in the uric acid levels in patients with and without diabetes (p for interaction in multivariable analysis <0.0001). Adjusted for multiple variables, each 1 mmol/L decrease in fasting serum glucose was associated with a 1.7 (± 0.3, p < 0.0001) μmol/L smaller fall in uric acid concentration in patients with diabetes. Conversely, each 1 mmol/L decrease in fasting serum glucose was associated with a 3.7 (± 1.5, p = 0.01) μmol/L larger fall in uric acid concentration in patients without diabetes.

### Urinary glucose and its influence on serum uric acid concentration

**Figure 4:**
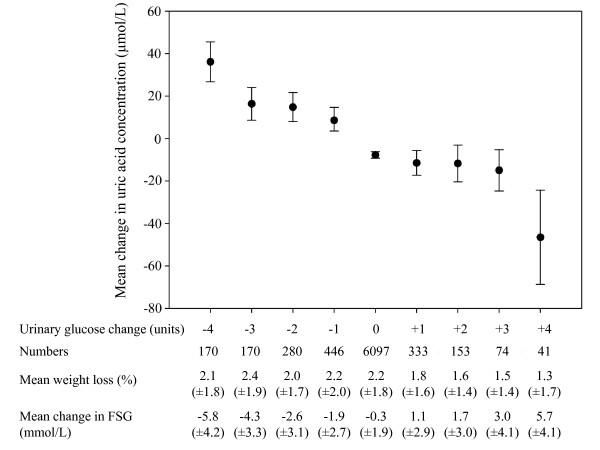
**Mean change in uric acid concentration for patients with diabetes, according to four week change in urinary glucose, estimated by dip stick**. The dip stick scale ranged between "negative", "trace", "1+", "2+" and "3+" for glucose content. FSG = fasting serum glucose. Error bars illustrate 95% confidence interval. Variables in table are presented as means (± standard deviation). Analysis for patients without diabetes was not performed, since 99% of the patients had a negative dip stick at screening, and 99% of the patients were found to have no change in glucose dip stick.

At the screening visit almost all patients without diabetes (99%) and approximately 80% of the patients with diabetes had a negative dip stick for urinary glucose. However, those patients showing a change in urinary glucose, measured by dip stick, were found to significantly alter their uric acid levels, as illustrated for all patients with diabetes in Figure 4. When glycosuria increased during the first 4 weeks of treatment with sibutramine and lifestyle changes, uric acid levels tended to fall; however, levels actually increased progressively in those with less glycosuria.

## Discussion

The once-daily treatment with sibutramine plus diet and exercise for four weeks induced significant mean reductions in uric acid concentrations overall, and patients without diabetes had greater reductions than those patients with diabetes. However, the significant fall in uric acid in the subgroup of patients gaining/maintaining their initial weight suggests a specific uric acid lowering effect of sibutramine and the results from some previous placebo controlled studies of sibutramine agree with this hypothesis with greater falls in uric acid for the same weight loss in sibutramine compared with placebo treated patients[13-15].

The present study found the response of weight loss and falling serum glucose levels on uric acid levels to be strongly modified by diabetes. In patients without diabetes weight loss had no detectable influence on uric acid concentrations and decreasing serum glucose concentrations were associated with decreasing uric acid levels. In patients with diabetes weight loss were found to have a paradoxical effect on the fall in uric acid concentrations - those with no weight loss and limited changes in blood glucose had the greatest uric acid reductions whereas those with maximum weight loss had negligible falls.

### Physiological mechanisms involved in uric acid metabolism

Renal elimination is the single most important factor regulating blood uric acid levels[16]. Uric acid is freely filtered through the renal glomerulus and uric acid concentration in blood is primary regulated by the degree of uric acid reuptake. Several transporters situated in the proximal tubule participate in uric acid reabsorption, and normally only 10% of the filtered load is excreted[17]. The most important uric acid transporter in the apical membrane has been considered to be the URAT1 transporter, which is thought to act in combination with two sodium-coupled monocarboxylate transporters (SMCT1 and SMCT2), as a "urate transportsome"[17]. This complex is influenced by a wide range of factors, such as inorganic and organic anions and several uricosuric drugs[17]. The uric acid is thought to then leave the tubule cell via the recently described SLC2A9 transporter, previously designated as the Glut9 transporter[18,19]. Several drugs have recently been described as inhibiting the SLC2A9 transporter and thereby increasing uric acid excretion and decreasing uric acid blood levels[18]. Potentially, sibutramine may be simulating these other drugs.

### SLC2A9, uric acid and glucose

The SLC2A9 transporter seems to be comprised of two splice variants: the Glut9 transporter, which is situated in the basolateral membrane, and the Glut9ΔN, which is situated in the apical membrane[20]. To our knowledge, two in vitro studies have investigated the characteristics of the SLC2A9 transporter[18,19]. One of the two studies found uric acid transport to be modestly accelerated by extra-cellular glucose, with the mechanism being classical exchange trans-stimulation between uric acid and glucose or fructose[19]. Importantly, this study seems to investigate both splice variants at the same time[19]. The other study focuses on the basolateral variant and found no stimulation of uric acid transport rate by extracellular glucose[17]. Hence, it seems that the exchange between uric acid and glucose is limited to the apical situated uric acid transporter.

### Differential effects of decreasing serum glucose levels on uric acid concentrations in diabetes

The different serum uric acid responses to decreasing levels of fasting glucose in patients with and without diabetes have been previously described with Chinese adults without diabetes also showing higher uric acid levels with higher fasting blood glucose whereas the opposite effect was seen in those with newly diagnosed diabetes[21].

The present study demonstrated that a decrease in serum glucose concentration was associated with a decrease in uric acid levels in patients without diabetes (Figure 3). Given that this patient group had cardiovascular disease and a relatively high fasting glucose (6.0 [±1.0] mmol/L) it is likely that these patients were insulin resistant with hyperinsulinaemia. As they lost weight however, serum insulin levels may have improved. Since insulin concentrations are inversely related to the urinary uric acid clearance[22,23], and infusing insulin has been shown to enhance coupled sodium and uric acid reabsorption[24], decreasing insulin levels might have limited the reabsorption of uric acid thereby helping to reduce blood uric acid levels. Patients with diabetes could be expected to experience a similar trend. However, patients with diabetes also display higher fasting glucose levels and are recognized to have much larger post-prandial blood glucose surges with greater glucose loading of the kidneys. Figure 4 presents the most direct evidence of the interactions between the renal handling of glucose and the renal mechanisms controlling blood uric acid levels. Thus, when there was little change in fasting glucose there was minimum change in the renal excretion of glucose and only about 10 μmol/L lower blood uric acid levels during the weight loss period whereas a deterioration in their diabetes control with an increase in fasting glucose (with probably higher insulin levels) was linked to a greater spillover of urinary glucose and the most marked fall in blood uric acid levels seen in the whole population group. This effect is the opposite of that expected from changes in insulin unless the insulin-mediated mechanism for uric acid reabsorption also displays progressive impaired effectiveness as well as in normal body glucose disposal. Those who reduced both their fasting and urinary glucose would be expected to lower their plasma insulin levels which would reduce renal uric acid reabsorption and lower blood uric acid concentrations. Yet the blood uric acid levels responded in the opposite direction by increasing markedly in this group and the range of urinary glucose-related responses in blood uric acid changes displayed almost a 100 μmol/L range. The recognized properties of the apical situated SLC2A9 transporter, i.e. exchanging intracellular uric acid for tubular glucose, seems therefore to be the explanation for the different responses: the greater load of glucose to the kidney in patients with diabetes is likely to facilitate uric acid excretion thereby markedly reducing blood uric acid levels as the diabetes state deteriorates.

The paradoxical relationship found between weight loss and uric acid levels in patients with diabetes is assumed to be secondary to the previously discussed differences in response of uric acid to falling serum glucose levels. However, it cannot be excluded that some of the inverse relationship between uric acid decrease and weight loss also potentially could be explained by factors like increased cell catabolism, as uric acid has previously been shown to increase during fasting[25].

### Other factors influencing uric acid concentrations

The relationship of uric acid levels to a change in HDL and LDL cholesterol seems of minor significance but the use of fibrates did seem to amplify the falls in serum uric acid. Multivariable analysis suggested that fibrates reduce by about a further 10 μmol/L the fall in uric acid observed over the four week period. Previous studies have shown a uric acid lowering effect of fenofibrate, with suggested mechanisms linked to increased urinary uric acid excretion[26-28], but the transporters involved are currently unclear. The converse effect applies to diuretics with increases in blood uric acid levels but hydrochlorthiazide has been found not to affect the basolateral membrane variant of SLC2A9 uric acid transport in vitro[18]. It is possible that induced uric acid reabsorption by diuretics counteracted the suggested sibutramine effect in some way. None of the other drugs included in the analysis (aspirin, ACE-inhibitors, calcium channel blockers or statins) was shown to have an influence on the change in uric acid concentrations.

### Limitations of the study

The present study was based on data obtained during four weeks of treatment with sibutramine, diet and exercise. Because fat masses and body fluids may have changed dynamically during this period, any observed relationship between uric acid, sibutramine, serum/renal glucose, and diabetes should be regarded as hypothesis-generating only. The lead-in period was non-randomized, single-blind and had no control group; therefore a direct uric acid lowering effect of sibutramine could not be isolated in this study, but has been demonstrated in previous placebo-controlled trials[8].

There is also a possibility that the results were affected by regression towards the mean or other factors. In particular, the higher screening values of uric acid among patients with CVD compared to that in patients without CVD could potentially account for some of the difference found in uric acid decrease between patients with and without diabetes. However, the fact that the group with CVD only and the group with DM+CVD had comparable screening values of uric acid but differences in decrement over the study period suggests that other factors were of importance. In addition, baseline values of uric acid were included in multivariable regression analysis.

Furthermore, measurements of insulin concentrations were lacking and their measurement would have helped in the interpretation of the data. On the other hand, serum insulin levels in patients with diabetes can be variable depending on the length of time with the disease, the type of therapy and the presence of differing degrees of insulin resistance. Also glycated haemoglobin could have been helpful in the interpretation of data; however, due to the design of the study and run-in period, there was not data available to permit meaningful analysis.

Anti-gout medication was not recorded in the study, but since nearly all uric acid values were within the normal range, the influence of such medication can be assumed to be minor. In addition genetic variation of the renal SCL2A9 transporter for uric acid transporters is now recognised to account for some of the variation in uric acid concentrations[19]. However, no such information on the genetic profiles of the SCOUT population is as yet available.

## Conclusion

A four week daily intake of sibutramine and life style changes was associated with a significant reduction in mean uric acid levels but the renal glucose load in patients with diabetes tended to counteract any selective uricosuric effect of sibutramine. The uric acid reduction during this short period of weight management was more pronounced in patients without diabetes.

## Competing interests

The SCOUT Executive Steering Committee comprises WPTJ (Chair), IDC, WC, LVG, NF, AM, CTP and AMS. RAR is employed by Abbott Laboratories.

## Authors' contributions

CA, PW, ELF, BB, LK and CTP analyzed the data for the present paper. CA wrote the initial draft of the manuscript. All authors interpreted the data and revised the manuscript.
